# Hypersensitivity Responses in the Central Nervous System

**DOI:** 10.3389/fimmu.2015.00517

**Published:** 2015-10-07

**Authors:** Reza Khorooshi, Nasrin Asgari, Marlene Thorsen Mørch, Carsten Tue Berg, Trevor Owens

**Affiliations:** ^1^Department of Neurobiology Research, Institute for Molecular Medicine, University of Southern Denmark, Odense, Denmark; ^2^Department of Neurology, Vejle Hospital, Vejle, Denmark

**Keywords:** antibody, complement, neuroinflammation, multiple sclerosis, neuromyelitis optica, autoantibody, central nervous system

## Abstract

Immune-mediated tissue damage or hypersensitivity can be mediated by autospecific IgG antibodies. Pathology results from activation of complement, and antibody-dependent cellular cytotoxicity, mediated by inflammatory effector leukocytes include macrophages, natural killer cells, and granulocytes. Antibodies and complement have been associated to demyelinating pathology in multiple sclerosis (MS) lesions, where macrophages predominate among infiltrating myeloid cells. Serum-derived autoantibodies with predominant specificity for the astrocyte water channel aquaporin-4 (AQP4) are implicated as inducers of pathology in neuromyelitis optica (NMO), a central nervous system (CNS) demyelinating disease where activated neutrophils infiltrate, unlike in MS. The most widely used model for MS, experimental autoimmune encephalomyelitis, is an autoantigen-immunized disease that can be transferred to naive animals with CD4^+^ T cells, but not with antibodies. By contrast, NMO-like astrocyte and myelin pathology can be transferred to mice with AQP4–IgG from NMO patients. This is dependent on complement, and does not require T cells. Consistent with clinical observations that interferon-beta is ineffective as a therapy for NMO, NMO-like pathology is significantly reduced in mice lacking the Type I IFN receptor. In MS, there is evidence for intrathecal synthesis of antibodies as well as blood–brain barrier (BBB) breakdown, whereas in NMO, IgG accesses the CNS from blood. Transfer models involve either direct injection of antibody and complement to the CNS, or experimental manipulations to induce BBB breakdown. We here review studies in MS and NMO that elucidate roles for IgG and complement in the induction of BBB breakdown, astrocytopathy, and demyelinating pathology. These studies point to significance of T-independent effector mechanisms in neuroinflammation.

## Introduction

Evolution and function of the immune system in mammals are driven by the need for protection against pathogenic infection. The balance between the conflicting requirements for capacity to recognize a universe of continually evolving microorganisms while avoiding deleterious responses to self poses a challenge. Hypersensitivity responses are defined as disorders that are caused by the immune response and include autoimmune responses. Three of the four classically described types of hypersensitivity involve antibodies. Type I hypersensitivity involves IgE antibodies and atopy and will not be discussed further. Types II and III hypersensitivity involve IgG antibodies and are implicated in immune pathology, especially the Type II hypersensitivity response, which explicitly involves autospecific antibodies. Type IV hypersensitivity involves T cell response, particularly CD4 T cell responses.

Generation of the self-specific antibodies that underpin the Type II hypersensitivity response occurs during B cell development when IgH V, J, and D segments recombine with junctional diversity, as well as IgL V–J recombination, resulting in over 10^11^ potential specificities. Selection against self-recognition occurs via IgL receptor editing as well as deletion via apoptosis. Nonetheless, B cell receptors with specificity for autoantigens persist in the adult repertoire. Protection against autoimmunity relies on a number of regulatory mechanisms, including the requirement for T cell help to generate a high affinity isotype-switched antibody response and that T cell activation is under separate and complex control. Autoantibodies may contribute to clearance of debris and effete cells as part of physiologically normal function, and so may not always be intrinsically deleterious. The T cell response that is required for IgG isotype switching does not itself need to be autospecific, since B cells may present innocuous or protective cross-reactive epitopes for T cell help (e.g., Molecular Mimicry). Nevertheless, it is clear from the fact of antibody-mediated autoimmune diseases that self-specific B cell clones can become activated and undergo isotype switching, with deleterious consequences. The degree to which this plays a role in neurological disease is of interest here.

Multiple sclerosis (MS) and neuromyelitis optica (NMO) are both autoimmune inflammatory demyelinating diseases in the central nervous system (CNS). The cause of MS is unknown, but multiple factors are considered to be involved in pathogenesis of MS. These include antibody-dependent mechanisms that contribute to the demyelination observed in Pattern II lesion pathology (1). Key features of Type II hypersensitivity that are relevant to discussion of their role in MS are specificity for tissue antigens (therefore autospecificity), recruitment of effector leukocyte responses, and activation of complement. In NMO, autoantibody binding to aquaporin-4 (AQP4) causes inflammation, astrocyte damage, cytokine release, and demyelination (2).

This review will deal with the role of Type II hypersensitivity autoantibody-driven responses in inflammatory demyelinating disease, with particular relevance to MS and NMO.

## Autoantibody in MS

Detection of IgG oligoclonal bands (OCB) in the cerebrospinal fluid (CSF) is one of the clinical criteria supporting the diagnosis of MS (3). CSF OCB occur in more than 90% of MS patients (4). Other isotypes, such as IgM and IgA, can also be found in CSF OCB (5, 6). Intrathecal IgM synthesis, presumed to be T cell independent, has been detected in 55% of MS patients (7, 8). OCB and polyspecific production of antibodies against measles, rubella, and varicella zoster virus, the so-called “MRZ reaction,” is associated with increased risk of converting from clinically isolated syndrome to MS (9). Possible involvement of antibodies in MS pathogenesis is suggested by beneficial response to therapeutic plasma exchange in MS patients retrospectively identified as having Pattern II lesions (10). However, it is important to note, that treatment with CD20-directed B cell-depleting therapeutics reduced the relapse rate in MS patients without affecting the presence of antibodies in the CSF (11).

Multiple sclerosis lesions have been classified on the basis of pathological patterns. Pattern II lesions are defined by presence of antibodies and activated complement product deposition. These lesions have been described in over 50% of actively demyelinating MS lesions (1). The specificity of the autoantibodies in MS remains largely unknown. MS lesions are mainly found in the CNS white matter, so one might expect candidate autoantibodies to be directed against antigen structures within this region. In MS lesions, autoantibodies against the potassium channel KIR4.1, myelin oligodendrocyte glycoprotein (MOG) and myelin basic protein (MBP) have been identified (12–15). However, consensus is lacking whether these autoantibodies are of pathogenic significance in patients with MS. A number of studies report failure to detect KIR4.1-specific IgG in serum or CSF from all but a fraction of patients with MS (16). Antibodies to MBP, although detectable, are not considered a meaningful biomarker for MS, since they have also been shown to be increased in response to neuronal damage (17).

The occurrence of pathogenic anti-MOG Ab is very rare in adult MS patients (18–20). Recent studies have described that anti-MOG Ab is detected in pediatric MS, ADEM (21, 22), and now in AQP4 seronegative NMO patients (23–27). During the progression of pediatric MS, epitope spreading can increase the number of CNS-reactive antibodies (28). This process of epitope spreading can be driven by antigen-presenting cells that present products of antibody-mediated breakdown of myelin and axonal specific antigens to T cells in the CNS (28).

Some earlier confusion about anti-MOG IgG in MS derived from use of assay techniques, such as ELISA and Western Blot, which detected antibodies that recognize incorrectly folded and denatured MOG, and therefore did not necessarily recognize MOG expressed in the CNS. Implementation of techniques, such as cell-based and tetramer assays (29), has improved discrimination of pathogenic antibodies and B cells, and, for example, allowed demonstration that axopathic and/or demyelinating autoantibody responses can occur in some patients with MS (30). However, anti-MOG antibodies are not considered to play a major role in adult MS, and at this time, no serum antibody specificity in adult MS is considered to be of diagnostic value. This leaves unanswered the question of what are the antigen specificities in OCB and what is their role in MS. Lipids have been identified among the autoantigens for OCB antibodies (31) and one study showed that lipid-specific oligoclonal IgM antibodies, especially for phosphatidylcholine, were prognostic for aggressive evolution of MS (32).

The animal model experimental autoimmune encephalomyelitis (EAE) can be induced by immunization with different myelin peptides, e.g., from MBP and MOG. This model is generally considered to be a T cell-mediated disease and cannot be transferred with antibodies (33). Nevertheless, co-transfer of IgG specific for MOG converted a non-demyelinating uniphasic EAE in Lewis rats to a relapsing–remitting demyelinating disease (34, 35).

Choice of antigen is highly influential when inducing EAE in mice (36). MOG–peptide-induced EAE has been shown to have no requirement for B cells, as it can be induced in animals without B cells (36, 37). Depletion of B cells exacerbated the clinical score in p35–55 MOG-induced EAE (38) indicating a regulatory role for B cells in EAE. On the other hand, MOG-specific TCR-transgenic mice that also expressed autoantibodies against MOG showed an accelerated and exacerbated course of EAE (39). Immunization of these or non-transgenic mice with human recombinant MOG extracellular domain or a fusion protein of MBP and proteolipid protein (MP4) both induced EAE, where activated B cells and antigen-specific antibodies played a pathogenic role in association with T cell-mediated inflammation (36, 40–43). Antigen-independent B cell infiltration and ectopic germinal center formation have been shown in mice with EAE induced by immunization with either a fusion protein incorporating the extracellular domain of mouse MOG or p35–55 peptide, both being T cell dependent (44). Thus, antibodies and B cells have a role to play in the animal model for MS, though the specific role is dependent on immunization strategy.

## Autoantibody in NMO

In NMO, disease-specific NMO–IgG (primarily of the IgG_1_ subclass) is a biomarker. The predominant NMO-associated antibody specificity is for the water channel AQP4 (45). AQP4 is densely localized in membranes of ependymal cells and astrocytes, to form the glia limitans of blood–brain barrier (BBB) and the CSF–parenchymal barrier (46). NMO–IgG/AQP4–IgG is thought to mediate pathogenesis by binding selectively to AQP4 on CNS astrocytes, causing complement fixation, generation of chemotactic signals (e.g., C3a, C5a), immune cell infiltration, and subsequent loss of AQP4 and glial fibrillary acidic protein (GFAP) on the astrocytes (2). Lesions in NMO are frequently found in the optic nerve and the spinal cord central gray matter as optic neuritis and transverse myelitis; however, brain lesions are also found at other sites of high AQP4 expression, such as the circumventricular organs (47–50). NMO–IgG is pathogenic only when reaching the CNS parenchyma as demonstrated in experimental animal studies where direct administration of NMO–IgG into the CNS or into the blood in mice with pre-established CNS inflammation-induced NMO-like histopathology, whereas peripheral administration into naïve animals had no effect (47, 51). In line with this observation, AQP4–IgG may exist for years after the first NMO attack without a relapse (52).

Other reported autoantibodies in NMO include anti-MOG as mentioned above (23–27), NMDA-type glutamate receptor (e.g., CV2/CRMP5), and glycine receptor antibodies (53–55). These and other autoantibodies may be useful biomarkers for NMO. However, their pathogenic importance has not been clarified. Future studies are required to establish this.

## Leukocytes in MS and NMO

Although the distribution of actively demyelinating lesions differs between MS patients, they are predominantly found within the optic nerves, spinal cord, brainstem, and periventricular white matter of the cerebral hemispheres (56). It has become clear in recent years that gray matter is not spared, even during the earliest phases of MS. Gray matter lesions show demyelination, neuronal loss, and atrophy (57–59). Gray matter lesions can be localized in or around the cortical and subcortical gray matter (60).

Inflammation is seen in both white and gray matter lesions at different stages of disease. It consists mainly of T-lymphocytes with a dominance of CD8^+^ T cells. However, B cells and plasma cells are also found in lesions. Macrophages are mainly found in white matter lesions, where they phagocytose myelin (56). The infiltration of T and B cells in CNS lesions was more profound in relapsing MS compared to progressive MS (61). Although the global composition of inflammatory cells is similar between relapsing-remitting and progressive disease (61), the relative number of plasma cells is higher in the progressive phase (61, 62). Clonally expanded B cells are detected in the CSF (63), in the meningeal lymphoid follicles, as well as in the parenchymal infiltrates in MS patients (64–66). A high ratio of B cells to monocytes in the CSF determined by flow cytometry correlated with rapid MS progression (67). Furthermore, lesion activity on MRI correlated with the numbers of plasmablasts in the CSF (68). These findings support a role for B cells in MS pathology.

Comparing the inflammation in MS lesions with NMO lesions, several studies have found that while the infiltrating cells in MS mostly consist of mononuclear cells, such as macrophages and T cells, inflammation in NMO include neutrophils, eosinophils, and mononuclear cells (2, 69, 70). These infiltrating cells, in particular macrophages, are implicated in Type II hypersensitivity through antibody-dependent cell-mediated cytotoxicity (ADCC).

The role of neutrophils and eosinophils in NMO pathology has been studied in animal models where NMO patient autoantibodies have been transferred to the CNS of mice to induce such pathology (51, 70, 71). When mice were made neutropenic, neuroinflammation was greatly reduced at 24 h and 7 days following intracerebral injection of patient autoantibodies (70). The fact that this had no effect on complement activation identified distinct modes of antibody effect. Neutropenic mice did not show loss of AQP4 or myelin, whereas intracerebral injection into neutrophil-enriched mice increased the areas of AQP4 and myelin loss and the number of inflamed cerebral vessels, thereby showing a role for granulocytes in tissue damage (70). Consistent with this, other studies showed that administration of a neutrophil protease inhibitor decreased the loss of AQP4 and myelin (70). Note that these studies would exclude either microglia or macrophages as mediators of pathology, since those cells should not have been affected by manipulations leading to neutropenia.

Eosinophils and neutrophils infiltrated NMO lesions in mice, after continuous infusion of patient autoantibodies (72). These granulocytes correlated to increased lesion size and both ADCC and complement-dependent cell-mediated cytotoxicity (CDCC) were involved (72). In addition, inhibition of eosinophil degranulation protected against ADCC and CDCC (72). Organotypic slice cultures were used to analyze synergy between antibody and leukocytes in induction of pathology. These transwell-based vibratome tissue slices from spinal cord, optic nerve, or hippocampus allowed analysis of an intact neuronal–glial network *in vitro*, and of effects of complement or leukocytes independently of infiltrating blood-derived cells or mediators. Pathology was complement dependent and under circumstances of suboptimal NMO–IgG, could be enhanced by addition of leukocytes, or pro-inflammatory cytokines (73), or eosinophils or their granule toxins (72). These studies also indicated that granulocytes play a role in formation of NMO lesions through both ADCC and CDCC. Addition of macrophages to slice cultures exacerbated pathology, dependent on complement, whereas natural killer (NK) cells caused loss of GFAP, AQP4, and myelin loss independent of complement (74). However, no evidence was found to support a role for NK cells in pathology in biopsy material from either MS or NMO patients (69), and granulocytes are the more likely effector cell.

Mechanism of action of pathogenic IgG in conjunction with leukocytes, or ADCC, involves Fc receptors (FcR). These are membrane glycoproteins expressed by leukocytes that have specific affinity for the Fc portions of immunoglobulin molecules, and thus link leukocytes via IgG to specific targets while signaling via the FcR. These are essential for a wide spectrum of biological activities, including transport of antibodies across cell membranes, induction of phagocytosis, and regulation of leukocyte function. Cross-linked FcR-bound antibody can initiate a signal transduction cascade that induces immune cell activation, resulting in cytokine production, immune cell proliferation, and degranulation of neutrophils, eosinophils, and mast cells (75, 76).

All of the effector mechanisms thus far described are components of the peripheral immune response. There is thus interest in the extent to which antibody entry from blood contributes to demyelinating pathology.

## BBB Integrity in Hypersensitivity Autoimmune Diseases in the CNS

Inflammation during disease activity in MS and NMO is frequently associated with BBB leakage, suggesting infiltration of the brain by inflammatory cells or immunoglobulin entering the CNS from the circulation (77). Studies of lesion pathology suggest that inflammation drives demyelination and neurodegeneration in MS patients (78). The BBB disruption in MS is primarily caused by infiltration of T cells responding to augmented expression chemokines and adhesion molecules at the luminal vascular endothelium, leading to migration of macrophages and dendritic cells, further increase of BBB permeability and leakage of inflammatory cytokines in the CNS to amplify the cascade of events (61). Complement components generated via the complement cascade are implicated in altered BBB permeability, further promoting inflammatory cell recruitment and Ig extravasation. Importantly, there can also be a role for antibody in BBB breakdown.

The BBB may be impaired before the occurrence of demyelinating foci and T-cell infiltration around small vessels (78). Disturbance of the BBB can be visualized by magnetic resonance imaging (MRI) through leakage of the magnetic marker gadolinium (Gd) diethylenetriamine pentaacetic acid (contrast enhancement) (79–81). An abnormal intra-BBB IgG synthesis rate was reported to correlate to the total area of MRI abnormality in the cerebrum (82). Elevated CSF/serum albumin ratio is evidence of BBB damage (83, 84). MS lesions are characterized by centrally placed inflamed veins, and fingerlike extensions of periventricular lesions (so-called Dawson’s fingers) (78). Collectively, the diagnostic implications of intra-BBB IgG synthesis and formation of OCB are well-established in MS, but how intra-BBB IgG production influences BBB integrity is not known.

**Figure 1:**
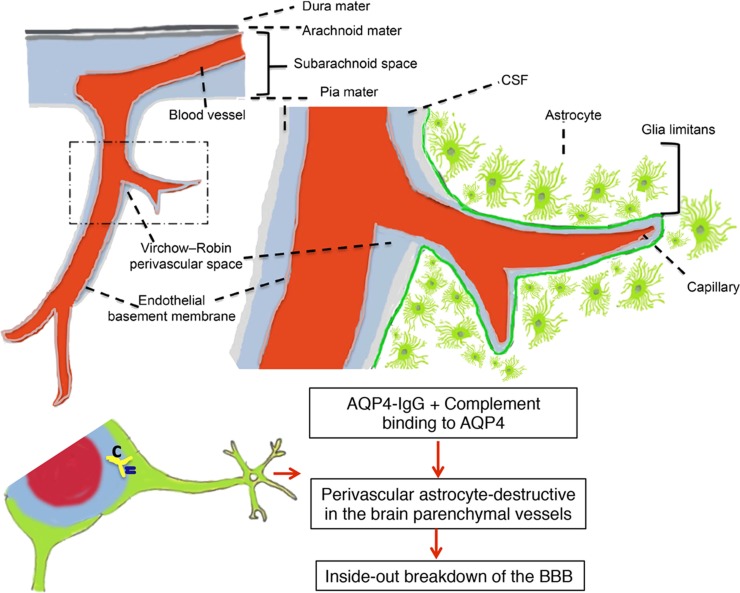
**Perivascular astrocyte-destructive lesions in the brain parenchymal vessels associated with breakdown of the blood–brain barrier**. Schematic presentation of subpial vasculature in relation to subarachnoid space and brain parenchyma showing relevant anatomical structures, including the pial vessel, subarachnoid space, the Virchow–Robin space, and the subpial glia limitans surrounding penetrating vessels into the brain. The intrathecal distribution pattern of aquaporin-4-immunoglobulin G from cerebrospinal fluid (CSF) into the brain parenchyma via a paravascular route leads to perivascular astrocyte-destructive lesions and blood–brain barrier breakdown [modified after Asgari et al. (90)].

A particular case in point that may help answer this question is provided by studies in NMO. Intrathecal AQP4–IgG is detectable in the CSF of the majority of AQP4–IgG seropositive NMO patients who have acute disease relapse with AQP4–IgG serum titers >1:250 (85, 86). The AQP4–IgG present in the CSF has been correlated with astrocyte damage, a primary pathological process in NMO (87, 88). Intrathecal IgG synthesis in NMO only occurs rarely and does not persist over time, and serum-derived AQP4–IgG is probably of major pathogenic importance (89). Taken together, these findings suggest entry of serum-derived AQP4–IgG to CNS during disease activity in NMO, which may further be deposited on astrocytic foot processes at the BBB, subpial, and subependymal regions. Thus, the destruction of the BBB may be an important step in the development of NMO because circulating AQP4–IgG has to pass through the BBB to reach the astrocytic endfeet, where AQP4 is localized. Astrocytes interact with endothelial cells to maintain the CNS BBB. We have very recently evaluated the pathogenic impact of AQP4–IgG in the CSF and find that intrathecal injection of AQP4–IgG together with human complement into the CSF of mice results in pronounced deposition of AQP4–IgG along subarachnoid space and subpial spaces, which initiated perivascular astrocyte-destructive lesions and consequently BBB breakdown (Figure 1) (90). These data suggest a model whereby a small amount of AQP4–IgG initially is spilled over to the CSF, and then initiates a pathogenic process, giving the characteristic CSF data and radiological features of human NMO. Thus, AQP4–IgG in CSF is a significant element in NMO pathogenicity and can be a critical element, which promotes perivascular astrocyte pathology and consequently BBB disruption. Whether these principles can apply to other antibody specificities, such as MOG-IgG, and to MS where there are intrathecal antibodies as well as BBB disruptions now become important questions.

Factors indicative of BBB integrity may serve as surrogate markers of NMO disease activity. Matrix metalloproteinase-9 (MMP-9) participates in the degradation of collagen IV, a major component of the cerebral vascular endothelial basement membrane (91), and of dystroglycan that anchors astrocyte endfeet to the basement membrane (92). MMP-9 is upregulated in MS lesions (93) and elevated serum levels of MMP-9 were reported in NMO and MS patients (91), interestingly higher in NMO than in MS (94), and likely increase BBB permeability in both diseases via effect on CNS microvascular endothelial cells. Intercellular adhesion molecule-1 (ICAM-1) and vascular cell adhesion molecule-1 (VCAM-1) play important roles in lymphocyte migration into the CNS. Higher levels of ICAM-1 and VCAM-1 have been reported in relapsing NMO patients and in MS compared to patients with non-inflammatory neurological disorders (95, 96). Furthermore, levels in NMO were higher than in MS and correlated with CSF albumin quotient (96). Another NMO marker of BBB breakdown, vascular endothelial growth factor-A (VEGF-A), has been implicated in promoting BBB breakdown in demyelinating disorders (97). Interestingly, an *in vitro* study demonstrated that AQP4–IgG binding to astrocytes alters AQP4 polarized expression leading to increased permeability of the astrocyte/endothelial barrier, reversed by application of an anti-VEGF-A blocking antibody, suggesting the potential role of VEGF-A in NMO pathology (98). Studies in AQP4 knock-out mice have highlighted important functional roles for AQP4 in the maintenance of BBB integrity as indicated by tight junction opening in brain microvessels, swelling of perivascular astrocytic processes, and BBB hyperpermeability (99). These data suggest that the pathogenic significance of serum-derived AQP4–IgG in NMO include BBB dysfunction. Whether astrocyte specificity of antibodies is required for analogous effects, in NMO as well as MS, is not known.

Central nervous system proteins are detected in sera and CSF of NMO patients, likely as part of compromised BBB and tissue damage. Neurofilament (NF) heavy chain levels have been implicated in optic neuritis associated with NMO, with high serum NF levels correlating with poor clinical outcome (100). In addition, astrocytic markers, including GFAP and S100B, are detected in the CSF in several inflammatory CNS disorders, including MS and NMO, and both are elevated in AQP4 IgG seropositive patients. CSF and serum levels of S100B correlated with active NMO disease, suggesting S100B may be a potential biomarker of acute relapse in seropositive NMO (87, 101).

Blood–brain barrier breakdown is thus a potentially important pathogenic element in inflammatory demyelinating diseases, and may be driven by antibodies as part of hypersensitivity processes in the CNS.

## Cytokines and Chemokines in Hypersensitivity Disorders in CNS

Cytokines and chemokines are involved in the control of inflammatory processes associated with demyelinating diseases in the CNS (102). They can be protective, but may also have deleterious effects. Changes in the microenvironment of the CNS following injury trigger an innate immune response, which involves germline-encoded pattern recognition receptors, such as toll-like receptors (103). These receptors recognize endogenous agonists released from damaged tissue as well as molecular patterns expressed by pathogens. This innate immune response includes induction of soluble products such as cytokines and chemokines that are critical for priming the antigen-specific adaptive immune response (104). Infiltrating cells and glial cells are both sources of cytokines and chemokines in the CNS.

Recruitment of leukocytes to tissue in hypersensitivity responses is driven by chemokines and by some cytokines. A number of studies support their involvement in NMO and MS, including that their levels in serum and CSF change dramatically compared to in healthy individuals. The role of inflammatory and anti-inflammatory cytokines in the pathogenesis of MS and in EAE has been broadly studied. Many of them have pathological and clinical significance in the context of autoantibody-mediated demyelination, although this has received less attention. Similarly, although the list of studies that have focused on cytokine and chemokine profiles in NMO is growing (105–110), there is still limited information about their functional significance in the pathogenesis of NMO.

Cytokines and chemokines that are classically implicated in recruitment and activation of B cells and leukocytes in a Type II hypersensitivity response would potentially include B-cell activating factor (BAFF), IL-1β, IL-6, TNFα, type I IFN, CXCL1/CXCL2 (and other CXCR2-binding chemokines), CXCL10 (IFN-induced protein-10), CXCL13 (B lymphocyte chemoattractant), CCL2 (macrophage chemotactic protein-1), and CCL11 (eotaxin). This is by no means a complete list but represents the principal candidate mediators that would be important in antibody-mediated pathology in MS and NMO. Evidence for their involvement is summarized in Table 1. Additionally, the role of selected entities, such as IL-1, IL-6, type I IFN, and certain chemokines, are separately discussed.

**Table 1:** **Cytokines, chemokines, and soluble mediators in CNS hypersensitivity**.

Mediator	Cell source	Role in hypersensitivity related process in CNS
BAFF	Astrocytes, leukocytes (111, 112)	Survival and maturation of B cells (111, 112)
IL-1	Microglia, astrocytes, neutrophils (113, 114)	Recruitment of leukocytes (115)
		Enhance C3 expression by astrocytes (116–118)
		T cell survival and effector functions (113)
IL-6	Microglia and astrocytes; virtually all immune cells (110, 119, 120)	Recruitment of leukocytes (120)
		Survival of plasmablasts, production of antibody (110)
TNFα	Microglia, astrocytes, and ependymal cells (121, 122)	Possible role in recruitment of leukocytes (122)
		Enhance C3 expression by astrocytes (117, 123)
		Cytotoxic for oligodendrocytes via TNFR1 cells (122)
Type I IFN	Glial cells, neurons, and leukocytes (124)	Proposed to reduce leukocyte migration across the BBB (124)
		Possible influence on complement induction (125, 126)
CXCL1	Astrocytes (127, 128)	Recruitment of neutrophils and T cells (129)
CXCL10	Astrocytes (121)	Recruitment of macrophages, neutrophils, and B cells (130)
ROS/RNS	Activated macrophages, granulocytes (131)	Influences leukocyte recruitment by affecting BBB permeability, and causing vasodilation (131)
		Cytotoxic to oligodendrocytes (131)
CXCL13	Microglia (132); follicular dendritic cells (133)	B cell recruitment (133)
		IgG affinity maturation (133)
CCL2	Glial cells, especially astrocytes (119)	Recruit monocytes through CCR2 (134)
		Promotes cytotoxic granule release by NK cells (135)
CCL11	Lymphocytes, macrophages, endothelial cells, and eosinophils (136–138)	Recruit eosinophils through CCR3 (137)
		Activation of basophils and T lymphocytes (136)

### IL-1

Increased levels of IL-1β have been reported in serum and CSF from MS and NMO patients (110, 139, 140). Increased expression of IL-1β by microglia/macrophages was detected in NMO patients with active lesions (characterized by AQP4 loss, astrocyte injury, immunoglobulin and complement deposition, and granulocyte infiltration). This likely depended on complement activation and granulocyte infiltration, since it was not shown in MS lesions or in advanced NMO lesions, which lacked complement activation and granulocyte infiltration (114). It was also shown that IL-1 enhanced formation of NMO lesions in spinal cord slice cultures treated with NMO–IgG and complement, but not in culture without NMO–IgG (73).

### IL-6

IL-6 levels in the CNS are normally undetectable, but increase during neuroinflammation, indicating their involvement in CNS diseases (141). Astrocytes and microglia are both sources of IL-6 (119, 141, 142). Elevated levels of IL-6 in the serum and CSF of NMO patients have also been reported (106, 140, 143). The severity of NMO–IgG and complement-induced lesions was increased when spinal cord slice cultures were treated with IL-6 (73). In another study, IL-6 was injected into the CNS of rats, and at the same time NMO–IgG was administered intraperitoneally. The results showed that IL-6 did not trigger formation of perivascular lesions with AQP4 loss distant from the needle track (114). Such findings suggest that IL-6 contributes to the pathogenesis of NMO as a secondary factor by facilitating the formation of NMO lesions. IL-6 also induces plasmablasts to produce autoantibody (144). IL-6 may also affect BBB integrity and has been implicated in BBB disruption (145, 146). All of these activities would potentially contribute to antibody-mediated pathology in MS and NMO.

### Type I IFN

Type I IFNs, including IFN-α and IFN-β, are known to play a crucial role in immune responses by activating JAK/STAT signals through their common receptor (IFNAR) (124). Unlike MS, IFN-β therapy has been reported to have very poor efficacy or to even exacerbate NMO (147) [reviewed in Ref. (124)]. IFN-β treatment in a NMO patient was associated with increased relapses and AQP4 antibody titers (147). Type I IFN signaling via the IFNAR receptor is required for NMO-like pathology in a mouse model (148). IFN-β therapy induced elevated serum levels of BAFF (111), which may facilitate autoantibody production in NMO (149). Elevated levels of IL-17, IFN-β, and neutrophil elastase were reported in serum from NMO patients, and the same study showed that IFN-β increased the formation of neutrophil extracellular traps (NETs) (150). Together, these findings suggested the severe exacerbation and increased relapses in NMO might be associated with IFN-β induced BAFF as well as degranulation and NETs formation by granulocytes (151). The fact that IFN-β had no effect on the development of NMO lesion in spinal cord slice culture, when it is treated with NMO–IgG and complement may reflect lack of neutrophil involvement (73). Lack of effect of IFNAR1-deficiency on cuprizone-induced de- and remyelination or glial cell response (152) may also reflect lack of neutrophil involvement.

## Cytokine Regulation of Complement in CNS

The complement system is an essential part of innate immunity and is important for protection against pathogens. The complement system is implicated in the pathogenesis of both MS and NMO (153, 154). Complement is activated by classical, alternative, and lectin pathways. All three pathways lead to activation of C3 convertases, release of C3b opsonin, C5 conversion, and, finally, membrane attack complex (MAC) formation. The activation of the complement pathway yields also C3a and C5a anaphylatoxins, potent inflammatory mediators, which target a broad spectrum of immune and non-immune cells. C3a and C5a are strong leukocyte chemoattractants, including neutrophils and B cells (75, 155). The classical pathway plays a major role in antibody-mediated pathology, and is activated when IgG or IgM antibodies bind to cell surface antigens.

Biosynthesis of complement in the human brain is reported to be generally low or non-detectable under normal health conditions (153). Complement activity presents a potent threat to the body’s own cells that are tightly protected by complement regulatory proteins, including decay-accelerating factor (DAF) and CD59. These complement regulatory proteins exist to protect the body’s own cells from damage caused by the activation of the complement pathway by blocking the formation of the C3 convertase and the MAC, respectively. DAF prevents the formation of C3 convertase by accelerating dissociation of C4b2a and C3bBb (classical and alternative C3 convertase). The complement regulator CD59 blocks the formation of the MAC by binding to C8, and thereby preventing further assembly of MAC. Therefore, the regulation of the expression of CD59 is a potentially important factor in protecting against MAC-mediated cytopathology (153). It has been shown that NMO–IgG and complement caused more severe longitudinally extensive spinal cord pathology in mice that lacked the complement regulator protein CD59 (156). However, the mechanism responsible for regulation of CD59 remains largely unknown.

Complement binding receptors are expressed on the surface of leukocytes and contribute to their response. The complement receptor 1 (CR1) is expressed on both neutrophils and B cells. It blocks the formation of C3 convertase by preventing its association with C2a. In addition, complement receptor 2 (CR2) participates with the B cell co-receptor complex in B cell activation. Complement receptors 3 and 4 (CR3 and CR4) are expressed on neutrophils and stimulate phagocytosis of bacteria and other particles that have complement components bound to their surface. CR3 is also important for leukocyte adhesion and migration processes (75, 153).

Complement was suggested to play a role in IL-6-induced CNS pathology (123). However, in contrast to IL-1β, IL-6 had no effect on the induction of complement by astrocytes in cell culture (157). The induction of complement seen in GFAP–IL-6 transgenic mice (123, 158), therefore, might not reflect the action of IL-6 alone, but rather of IL-6 acting in concert with other cytokines, including IL-1β. IL-1 is involved in regulation of complement component C3 in astrocytes (157, 159). Whether and how IL-1 influences complement-mediated astrocyte damage remains to be addressed.

Type I IFN can also influence complement in the CNS. The level of terminal complement complex, C1-inh, C4, and C3bc increased in IFN-α2a-treated MS patients during the initial part of the treatment (125, 126). It was shown that IFN-α and IFN-β, in a dose-dependent manner, stimulated the synthesis of C2, C1-inh, and factor B, but not C3 in human monocytes *in vitro* (160). It was earlier noted that IFN-α/β selectively stimulated the synthesis of factor B and C1 inh, but reduced C3, and had no effect on C2 (161). The results from these studies suggest the involvement of type I IFN in the induction of selective complement components, but how the increased complement level is directly mediated by IFNAR signaling was not determined. In antibody-mediated pathology, such as in NMO, where the complement system is known to play a significant role and there is evidence for the involvement of type I IFN, it is tempting to speculate that the induction of complement by type I IFN is one of the underlying mechanisms that facilitate the formation of NMO lesion.

## Regulatory Role for Microglia in Antibody-Mediated Pathology

Microglia are considered to play a critical role in regulation of inflammatory processes within the CNS. In this regard, IL-6 also exerts a protective function and has anti-inflammatory activities (162, 163). Administration of human rIL-6 dramatically reduced demyelination and inflammation, which was induced by TMEV in the spinal cord of mice (164). A fusion protein of the soluble IL-6 receptor to IL-6 (IL6RIL6) prevented neuronal and oligodendrocyte degeneration in organotypic hippocampal slices (165). This is in line with *in vivo* results that showed administration of IL6RIL6 to rats after sciatic nerve transection-stimulated remyelination (166) as well as -accelerated regeneration of axotomized peripheral nerve in transgenic mice expressing both IL-6 and IL-6R (167). However, IL-6-activated microglia produced NO, resulting in neural injury *in vitro* (168).

### Chemokine and cytokine involvement in leukocyte recruitment

The cytokine IL-6 is implicated in extravasation of leukocytes into the CNS (168–172). Injection of IL-1β into CNS caused the formation of perivascular lesions with granulocytic infiltration and AQP4 loss distant from the injection site (114), also suggesting a role for IL-1β in leukocyte extravasation. The level of CXCL10, a downstream chemokine of type I IFN signaling (173–175), is elevated in NMO (105, 109). Astrocytes (119) and neutrophils (176) both produce CXCL10. Although CXCL10 is primarily associated with recruitment of T cells, it can also induce neutrophil recruitment (177, 178). One mechanism by which type I IFN signaling exacerbates NMO may involve induction of CXCL10 and thereby recruitment of neutrophils.

## Concluding Remarks

**Figure 2:**
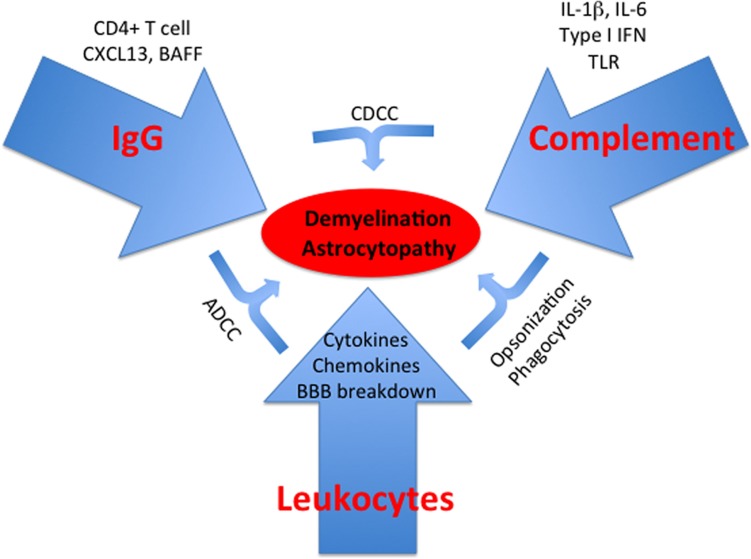
**Type II hypersensitivity responses in the CNS**. Schematic summarizing key aspects discussed in the text. TLR, toll-like receptor.

We have here reviewed evidence for a role for antibody-mediated hypersensitivity mechanisms in MS and NMO. It must be emphasized that these mechanisms do not normally occur in isolation from effector T cell-mediated responses, whether CD4^+^ or CD8^+^. Also, direct pathology mediated by activated leukocytes may also contribute along with the ADCC mechanisms that we have highlighted. Nevertheless, the studies that we have reviewed demonstrate that antibody and especially IgG are powerful mediators in neuroinflammation and that they must be given equal weight in consideration of design of therapies for MS and NMO (Figure 2).

## Conflict of Interest Statement

The authors declare that the research was conducted in the absence of any commercial or financial relationships that could be construed as a potential conflict of interest.

## Funding

Research in the Owens lab on which this review is based is funded primarily by the Danish MS Society, the Region of Southern Denmark, The Lundbeck Foundation, and the Danish Council for Independent Research (Health and Disease).
